# Mitochondrial dysfunction in airways and quadriceps muscle of patients with chronic obstructive pulmonary disease

**DOI:** 10.1186/s12931-020-01527-5

**Published:** 2020-10-12

**Authors:** Gulam Haji, Coen H. Wiegman, Charalambos Michaeloudes, Mehul S. Patel, Katrina Curtis, Pankaj Bhavsar, Michael I. Polkey, Ian M. Adcock, Kian Fan Chung, on behalf of the COPDMAP consortium

**Affiliations:** 1 Airways Disease, National Heart and Lung Institute, Imperial College London, Dovehouse Street, London, SW3 6LY UK; 2 Royal Brompton & Harefield NHS Foundation Trust, London, UK

**Keywords:** COPD, Airways, Quadriceps, Oxidative stress, Mitochondria

## Abstract

**Background:**

Mitochondrial damage and dysfunction have been reported in airway and quadriceps muscle cells of patients with chronic obstructive pulmonary disease (COPD). We determined the concomitance of mitochondrial dysfunction in these cells in COPD.

**Methods:**

Bronchial biopsies were obtained from never- and ex-smoker volunteers and COPD patients (GOLD Grade 2) and quadriceps muscle biopsies from the same volunteers in addition to COPD patients at GOLD Grade 3/4 for measurement of mitochondrial function.

**Results:**

Decreased mitochondrial membrane potential (ΔΨm), increased mitochondrial reactive oxygen species (mtROS) and decreased superoxide dismutase 2 (SOD2) levels were observed in mitochondria isolated from bronchial biopsies from Grade 2 patients compared to healthy never- and ex-smokers. There was a significant correlation between ΔΨm and FEV_1_ (% predicted), transfer factor of the lung for carbon monoxide (TLCO_C_ % predicted), 6-min walk test and maximum oxygen consumption. In addition, ΔΨm was also associated with decreased expression levels of electron transport chain (ETC) complex proteins I and II. In quadriceps muscle of Grade 2 COPD patients, a significant increase in total ROS and mtROS was observed without changes in ΔΨm, SOD2 or ETC complex protein expression. However, quadriceps muscle of GOLD Grade 3/4 COPD patients showed an increased mtROS and decreased SOD2 and ETC complex proteins I, II, III and V expression.

**Conclusions:**

Mitochondrial dysfunction in the airways, but not in quadriceps muscle, is associated with airflow obstruction and exercise capacity in Grade 2 COPD. Oxidative stress-induced mitochondrial dysfunction in the quadriceps may result from similar disease processes occurring in the lungs.

## Background

Chronic obstructive pulmonary disease (COPD) is characterised by a persistent, progressive airflow obstruction and increased chronic inflammation in the airways and lungs. The pathological features include emphysema, mucus hypersecretion, small airways inflammation and fibrosis, and increased airway smooth muscle (ASM) mass due to ASM cell proliferation, which lead to airflow obstruction. Patients with more advanced COPD experience frequent exacerbations caused by viruses and/or bacteria, associated with a faster decline in lung function and greater mortality [1, 2]**.** Oxidative stress derived both exogenously through cigarette smoking and endogenously through inflammatory processes is an important mechanism driving the damage and inflammation in COPD [1]. Markers of oxidative stress such as hydrogen peroxide and lipid peroxidation products such as 8-isoprostanes and malonaldehyde are increased, while antioxidants such as glutathione are reduced [2].

Mitochondria are the most important source of intracellular reactive oxygen species (ROS), generated during the synthesis of ATP through oxidative phosphorylation. Mitochondrial reactive oxygen species (mtROS) are maintained as a balance between production and scavenging processes from the antioxidant defence system. Elevated levels of mtROS can initiate processes that influence mitochondrial function and integrity.

Indeed, failure to scavenge mtROS leads to mitochondrial dysfunction as shown in airway epithelial cells exposed to cigarette smoke extract (CSE) leading to decreased ATP levels and mitochondrial membrane potential and impaired mitophagy [3–5]. In addition, CSE exposure leads to mitochondrial fragmentation, branching and reduced cristae linked to increased levels of IL-1β, IL-6, and CXCL8 that are associated with features of senescence [6, 7]. Similar changes of mitochondrial damage with fragmentation has been observed in airway epithelial cells obtained from patients with COPD, and altered mitochondrial function has been shown in airway smooth muscle cells cultured from biopsies obtained from patients with COPD as assessed by reduced membrane potential, respiration rates and ATP production [8].

One extra-pulmonary manifestation of COPD is locomotor skeletal muscle dysfunction, specifically atrophy and weakness of these muscles, and reduced muscle endurance described in the quadriceps muscle even in mild disease [9]. The impairment in muscle endurance observed in COPD is related to a reduced muscle oxidative capacity, together with reductions in both mitochondrial density and enzyme activities [10–12]. A recent study supported the concept that the low muscle oxidative capacity in COPD cannot be explained by physical inactivity alone and is likely driven by COPD pathophysiological processes in the lungs of patients with COPD [13], bringing in the concept that circulating mediators derived from the COPD lung could cause mitochondrial dysfunction in skeletal muscle particularly the quadriceps muscle.

We conducted this study in order to examine mitochondrial function from mitochondria isolated from bronchial biopsies and quadriceps muscle of patients with COPD compared to those obtained from smokers without COPD and to assess whether there were parallel changes in both compartments.

## Methods

### Study design and procedures

Non-smoking healthy and COPD participants were recruited from the Royal Brompton Hospital and enrolled through the Medical Research Council COPD MAP Consortium. COPD participants were enrolled with severity levels defined according to the Global initiative for chronic Obstructive Lung Disease (GOLD) guidelines. Participants with an exacerbation or symptoms of a respiratory tract infection in the preceding month were excluded. All participants were assessed at an initial visit, then undertook an incremental maximal cycle-ergometer cardiopulmonary exercise test (CPET) with cardiorespiratory measurements, a six-minute walk test and quadriceps strength measurements over two visits. For safety considerations, COPD participants at GOLD Grade 3/4 did not undergo bronchoscopy and only underwent a muscle biopsy. All participants gave informed consent to this study that was approved by the Research Ethics Committee (11/LO/1852 and 12/LO/0088).

### Fiberoptic bronchoscopy

Fiberoptic bronchoscopy was undertaken in participants under sedation, with midazolam and topical anaesthesia to the airways with lidocaine. Standard sized biopsy forceps were used to take four to six endobronchial biopsies from the right middle lobe and the right lower lobe bronchi. Biopsies were collected in cold PBS and transported on ice to the laboratory for protein fraction isolation.

### Quadriceps muscle biopsy

A percutaneous needle biopsy of the Vastus lateralis was obtained from the dominant side at least 1 week after but within 1 month of phenotypic characterisation. Under sterile conditions (2% chlorhexidine/70% isopropyl), 5 ml of 2% lignocaine was injected into the skin and subcutaneous tissue. A sub centimetre incision was made, and a Bergstrom needle inserted through the subcutaneous tissues and the fascia of the muscle. Several tissue biopsies (4–6, approximately 0.5 cm^2^) were collected at the same pass. Biopsies were collected in cold PBS and transported on ice to the laboratory for protein fraction isolation.

### Mitochondrial functional analysis

Protein fraction isolation from bronchial and quadriceps biopsies were performed immediately after obtaining the biopsy. Intact mitochondria and cytoplasmic protein fractions were isolated using a Mitochondrial Isolation Kit for Tissue (Thermo-Scientific) and a Dounce Tissue Grinder set (SIGMA). Briefly, to disrupt cell-to-cell contact bronchial and quadriceps biopsies were dounced with douncer A by 5 strokes and 10 strokes, respectively. This was followed by douncing with 20 and 40 strokes for bronchial and quadriceps biopsies, respectively, with douncer B to disrupt the cells. Cellular contents were separated into three different fractions by two centrifugation steps. The large nuclear membrane fraction was pelleted by centrifugation at 2500 rpm for 10 min at 4 °C. The supernatant containing the cytoplasm fraction with mitochondria was transferred to new tubes and centrifuged subsequently at 10,500 rpm for 15 min at 4 °C. The mitochondrial pellets were dissolved in Hanks’ Buffered Salt Solution (HBSS) containing MgCl_2_ and CaCl_2_ (Gibco). After isolation the protein content of all the fractions was determined by Bradford assay (Biorad) against a 2 mg/ml bovine serum albumin standard.

Mitochondrial membrane potential (ΔΨm) was measured in intact isolated mitochondria using the cationic dye 5,5′,6,6′-tetrachloro-1,1′,3,3′-tetraethylbenzimidazolylcarbocyanine iodide (JC-1, Invitrogen). Intact mitochondria were incubated with JC-1 (3 μM) for 30 min at 37 °C and 5% CO_2._ JC-1 monomers emit green fluorescence but when they enter live mitochondria, they form J-aggregates which emit red fluorescence. The ratio between red and green fluorescence was used as an index of ΔΨm and is expressed per mg protein of each sample.

Mitochondrial reactive oxygen species (ROS) were measured with MitoSOX™ Red (Invitrogen), a redox-sensitive fluorescent probe that is selectively targeted to the mitochondria. Intact isolated mitochondria were incubated with 5 μM MitoSOX and the red fluorescence was determined at 510/580 nm using a fluorescence plate reader. Whole cellular ROS was detected in the cytoplasm fractions using the conversion of the cell-permeable non-fluorescent 2′-7′-dichlorofluorescin diacetate (DCF) probe into the highly fluorescent 2′-7′-dichlorofluorescin when de-esterified by oxidation products. The cytoplasmic fractions were incubated with 0.1 mM DCF for 30 min at 37 °C and 5%CO_2_. Fluorescence was measured at 485/528 nm on a SynergyTM multi-detection microplate reader (Biotek Instruments, USA). Data is expressed as relative fluorescence units (RFU) per mg protein.

Superoxide dismutase 2 (SOD2) levels in mitochondrial fractions were determined by sandwich ELISA (R&D systems) according to manufacturer’s instructions and are expressed per mg protein of each sample.

### Mitochondrial electron transport chain (ETC) complex proteins I, II, III, IV and V

Content of isolated protein fractions were determined by Bradford assay (Biorad). Mitochondrial protein fractions (20 μg per lane) were separated on 10% NuPAGE gels (Invitrogen) and proteins were detected as previously described [14]. Bronchial and quadriceps muscle mitochondrial electron chain transport (ETC) complex proteins were detected using the MitoProfile Total OXPHOS Human WB Antibody Cocktail (MitoScience). Porin (VDAC1) protein expression was used as a loading control (Abcam).

### Statistical analysis

Data involving patient details and assessments are expressed as mean ± SEM. Differences in continuous variables (Table 1) were assessed parametrically using ANOVA and t-tests after normality of the dataset was tested using the Kolmogorov-Smirnov test.

**Table 1:** Subject characteristics

	Never-smokers ***n*** = 5	Ex-smokers ***n*** = 8	GOLD Grade 2 ***n*** = 6	GOLD Grade 3/4 ***n*** = 6
**Age (years)**	61 ± 6	63 ± 5	64 ± 3	64 ± 7
**Female**: **Male**	1:4	3:5	4:2	1:5
**Cigarette smoking** **(pack years)**	0 ± 0	36 ± 16	34 ± 12	40 ± 6
**Body mass index (kg/m**^**2**^**)**	24 ± 5	25 ± 4	29 ± 7	23 ± 3 #
**CAT score**	2 (0–6)	8 (2–15)	16 (4–23)	24 (20–26)**#
**FEV**_**1**_ **(L)**	2.9 ± 0.4	2.9 ± 0.7	1.9 ± 0.3	0.8 ± 0.3**###
**FEV**_**1**_ **(% pred)**	100 ± 9	103 ± 11	71 ± 5*	30 ± 11**##
**FVC (L)**	4.3 ± 0.8	4.4 ± 1.0	3.0 ± 0.7	3.1 ± 1.3
**FEV**_**1**_**/FVC (%)**	69 ± 6	67 ± 7	63 ± 8	28 ± 9**##
**TLCOc (% pred)**	90 ± 14	80 ± 10	67 ± 8*	38 ± 13**##
**6-min walk (m)**	657 ± 18	631 ± 55	493 ± 66**	364 ± 65**##
**VO**_**2**_ **Peak (ml/kg/min)**	28 ± 3	25 ± 3	17 ± 3**	12 ± 4**#
**QMVC (Kg)**	34 ± 6	41 ± 11	31 ± 8	32 ± 6
**QMVC/BMI**	1.5 ± 0.1	1.6 ± 0.4	1.1 ± 0.3*	1.4 ± 0.2
**SPPB**	12	12 (11–12)	11 (10–12)	11 (9–12)

*BMI* Body Mass Index; CAT score: COPD Assessment Test score; *FEV1* Forced Expiratory Volume in one sec; 6 MW: 6-Minute Walk; *QMVC* Quadriceps Maximal Voluntary Contraction; *SPPB* Short Physical Performance Battery; *TLCOc* Single-breath transfer factor of the lung for carbon monoxide; *VO*_*2*_ Oxygen consumption. Data shown as mean ± SD or median (range). * *p* < 0.05 compared to never smoker or ex-smoker, **p < 0.01 compared to never smoker or ex-smoker, ^#^*p* < 0.05 compared to GOLD Grade 2, ^##^ *p* < 0.01 compared to GOLD Grade 2, ^###^ *p* < 0.01 compared to GOLD Grade 2

Pearson and Spearman’s correlations were determined between mitochondrial membrane potential (JC-1) and patient assessments (FEV_1_, TLCOc, 6 MW and VO_2_) and between JC-1 and the electron transport chain (ETC) complex protein expression levels in bronchial and quadriceps biopsies, respectively. Biopsy analysis was assessed non-parametrically by Kruskal Wallis ANOVA and Mann-Whitney test. *P* value of ≤0.05 was taken as significant.

## Results

### Subject characteristics

There was no difference in lung function and exercise performance between the healthy never-smokers and ex-smokers with an FEV_1_ > 80% predicted (Table 1). As expected, patients with GOLD Grade 3/4 disease demonstrated poorer exercise performance and higher symptom burden than those with GOLD Grade 2 disease and never-smokers or ex-smokers, together with a reduced peak oxygen consumption (VO_2_ Peak) and six-minute walk distance. GOLD Grade 2 patients also demonstrated a lower VO_2_ Peak and six-minute walk distance when compared to ex-smokers and never-smokers.

### Airway mitochondrial function, ROS and ETC complex protein levels

**Fig. 1:**
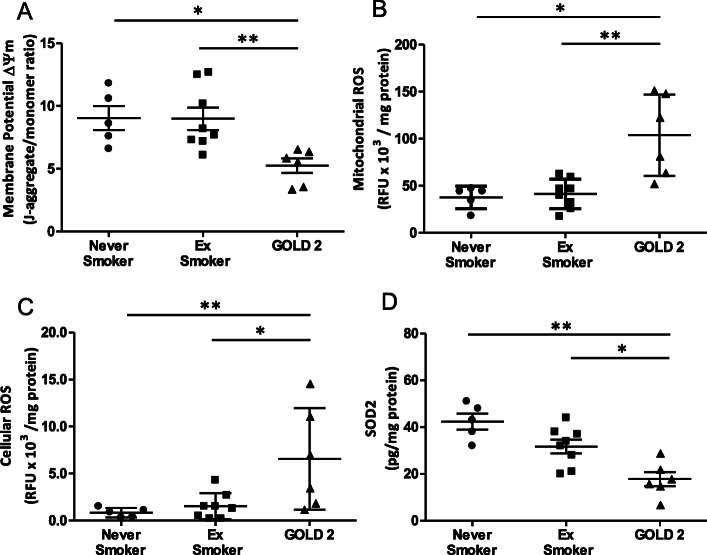
Mitochondrial function, ROS and ETC complex protein expression levels in the lung. (**a**) Airway mitochondrial membrane potential (ΔΨm), (**b**) mitochondrial reactive oxygen species (mtROS), (**c**) cellular ROS levels and (**d**) superoxide dismutase 2 (SOD2) levels in never-smoking healthy, ex-smokers and GOLD Grade 2 patients. Data is expressed as mean ± SEM; healthy never-smokers (*n* = 5), ex-smokers (*n* = 8) and GOLD Grade 2 patients (*n* = 6); *p < 0.05, **p < 0.01, Mann-Whitney test; RFU: Relative Fluorescence Units

Patients with GOLD Grade 2 COPD demonstrated a significant reduction in airway mitochondrial membrane potential (ΔΨm) when compared to ex-smokers (*p* < 0.01) or never-smokers (*p* < 0.05) (Fig. 1a), with higher levels of mitochondrial ROS when compared to healthy ex-smokers (*p* < 0.01) and healthy never-smokers (*p* < 0.05) (Fig. 1b). Overall cellular ROS burden was also higher in the GOLD Grade 2 patients when compared to the healthy never-smoker (*p* < 0.01) and ex-smoker (*p* < 0.05) groups (Fig. 1c). Superoxide dismutase 2 (SOD2) levels were decreased in GOLD Grade 2 COPD patient mitochondrial fractions compared to ex-smokers (*p* < 0.05) and never-smokers (*p* < 0.01) (Fig. 1d).

**Fig. 2:**
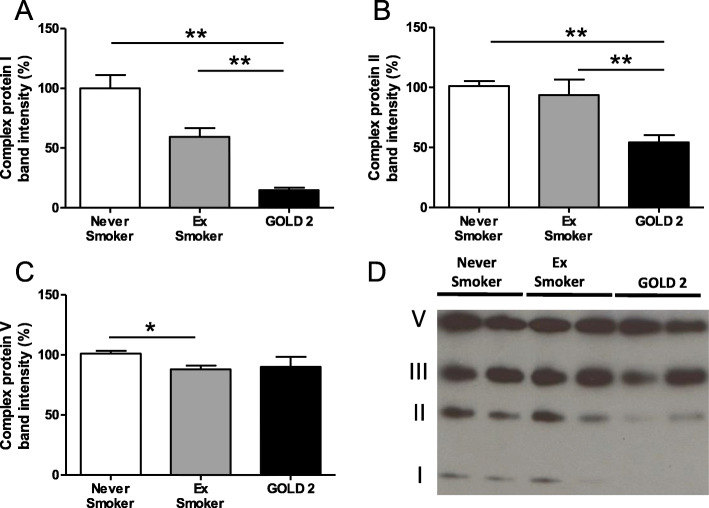
ETC complex protein expression levels in the lung. **(a**) Electron transport chain (ETC) complex protein I, (**b**) complex protein II, and (**c**) complex protein V expression levels. **(d)** Representative Western blot images from 2 never-smoker, 2 ex-smokers and 2 GOLD Grade 2 COPD patients of mitochondrial ETC proteins. Data is expressed as mean ± SEM; healthy never-smokers (*n* = 5), ex-smokers (*n* = 5) and COPD patients (*n* = 5). *P < 0.05 and **P < 0.01, Mann-Whitney test

ETC complex protein I (NADH dehydrogenase) protein expression was reduced in mitochondria isolated from COPD patient biopsies compared to healthy (− 85.1%, *p* < 0.01) and ex-smoker (− 42.2%, *p* < 0.01) groups (Fig. 2a). ETC complex protein II (ubiquinol-cytochrome c reductase complex) protein expression was also reduced in lung mitochondria isolated from COPD patient lung compared to healthy (− 45.8%, *p* < 0.01) and ex-smoker (− 39.4%, *p* < 0.01) groups (Fig. 2b). A small reduction in ETC complex protein V (ATPase) protein expression was observed in mitochondria isolated from the ex-smoker group lungs compared to the never-smoker group (− 13.9%, *p* < 0.05). No change in protein expression of the ETC complex V protein was observed in the COPD group. Representative Western blot images from 2 never-smoker, 2 ex-smokers and 2 GOLD Grade 2 COPD patients are shown in Fig. 2d.

**Fig. 3:**
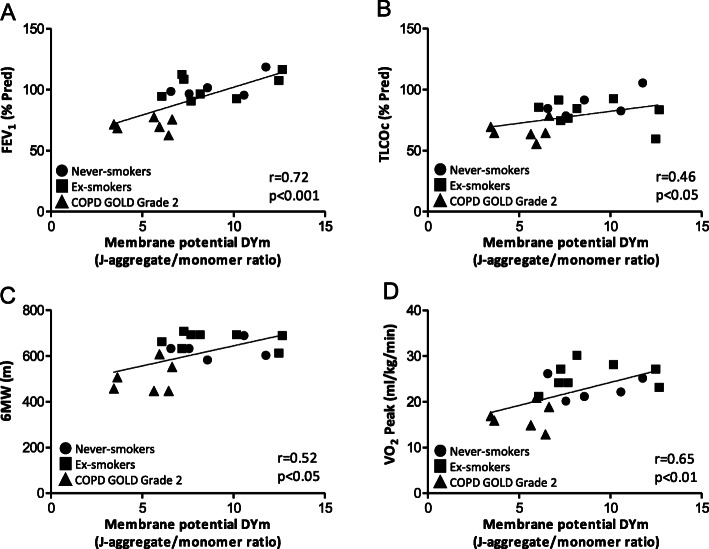
Correlations between airway mitochondrial membrane potential and FEV1, TLCOc and VO_2_ Peak. (**a**) Correlations between airway mitochondrial membrane potential (ΔΨm) and FEV_1_ (% predicted), (**b**) single-breath transfer factor of the lung for carbon monoxide (TLCOc % predicted), (**c**) six-minute walk distance (6 MW), and (**d**) weight-adjusted peak oxygen uptake (VO_2_ peak ml/min/kg). ● never-smokers (*n* = 5), ■ ex-smokers (*n* = 8) and ▲COPD GOLD Grade 2 (*n* = 6)

**Fig. 4:**
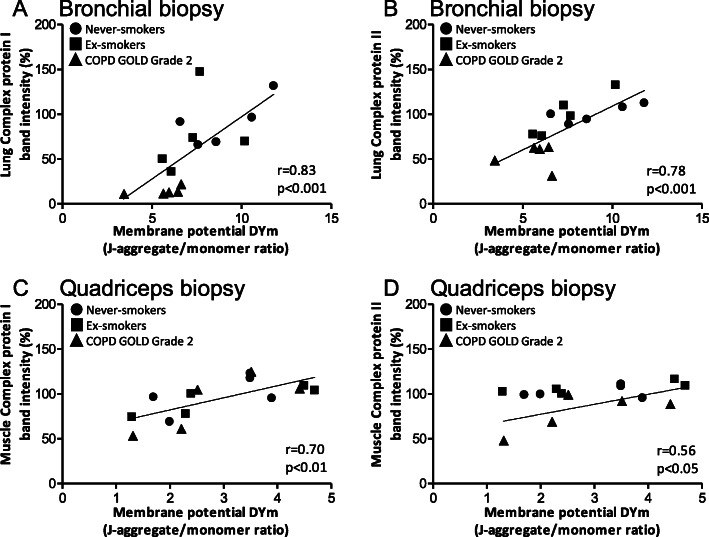
Correlation between mitochondrial membrane potential and ETC protein expression levels in quadriceps muscle. (**a**) Correlations between bronchial mitochondrial membrane potential (ΔΨm) and protein expression levels of lung electron transport chain (ETC) complex proteins I and (**b**) lung ETC complex protein II. (**c**) Correlation between quadriceps mitochondrial membrane potential (ΔΨm) and muscle ETC complex protein I and (**d**) muscle ETC complex protein II ● never-smokers (*n* = 5), ■ ex-smokers (*n* = 5) and ▲COPD GOLD Grade 2 (*n* = 5)

There was a significant correlation between airway mitochondrial membrane potential (ΔΨm) and FEV_1_% predicted (*r* = 0.72, *p* < 0.001), and TLCO_c_ % predicted (*r* = 0.46, *p* < 0.05) (Fig. 3a & b). Six-minute walk distance was also correlated with membrane potential (*r* = 0.52, *p* < 0.05) as was the peak oxygen consumption VO_2_ (*r* = 0.65, *p* < 0.01) (Fig. 3c & d). In addition, there was a correlation between ΔΨm and the level of ETC complex proteins I and II measured on the Western blots from bronchial biopsy mitochondria (*r* = 0.73; *p* < 0.001 and *r* = 0.79, *p* < 0.001, respectively; Fig. 4a & b).

### Quadriceps muscle mitochondrial function & ROS and ETC complex protein levels

**Fig. 5:**
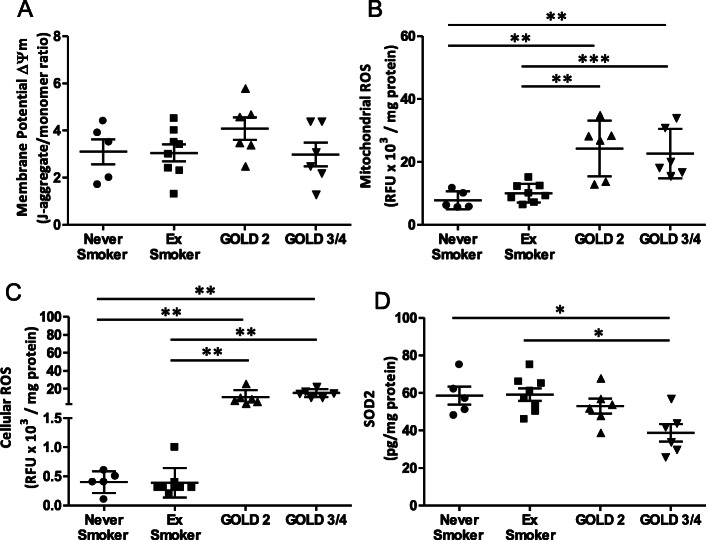
Mitochondrial function, ROS and ETC complex protein expression levels in quadricep muscle. (**a)** Quadriceps muscle mitochondrial membrane potential (ΔΨm), (**b**) mitochondrial reactive oxygen species (mtROS) levels, (**c**) total cellular ROS levels and (**d**) superoxide dismutase 2 (SOD2) levels in healthy never-smokers, ex-smokers, GOLD Grade 2 and GOLD Grade 3/4 patients. Data shown as mean ± SEM; never-smokers (*n* = 5), ex-smokers (*n* = 8), GOLD Grade 2 patients (*n* = 6), and GOLD Grade 3/4 patients (*n* = 6); *p < 0.05, **p < 0.01, Mann-Whitney test

There was no difference in ΔΨm in the quadriceps muscle between the study groups (Fig. 5a). There were higher whole cell and mitochondrial ROS levels in the quadriceps muscle of GOLD Grade 2 and GOLD Grade 3/4 patients when compared to the healthy never-smokers and ex-smoker groups (Fig. 5b & c). SOD2 levels were decreased in GOLD Grade 3/4 COPD patients compared to never-smokers (*p* < 0.05) and ex-smokers (*p* < 0.05) but not to GOLD Grade 2 COPD patients. There was no significant correlation between ΔΨm and ROS levels in quadriceps muscle with quadriceps muscle strength, FEV_1_ (%predicted), TLCOc (% predicted), six-minute walk distance and peak VO_2_ (data not shown).

**Fig. 6:**
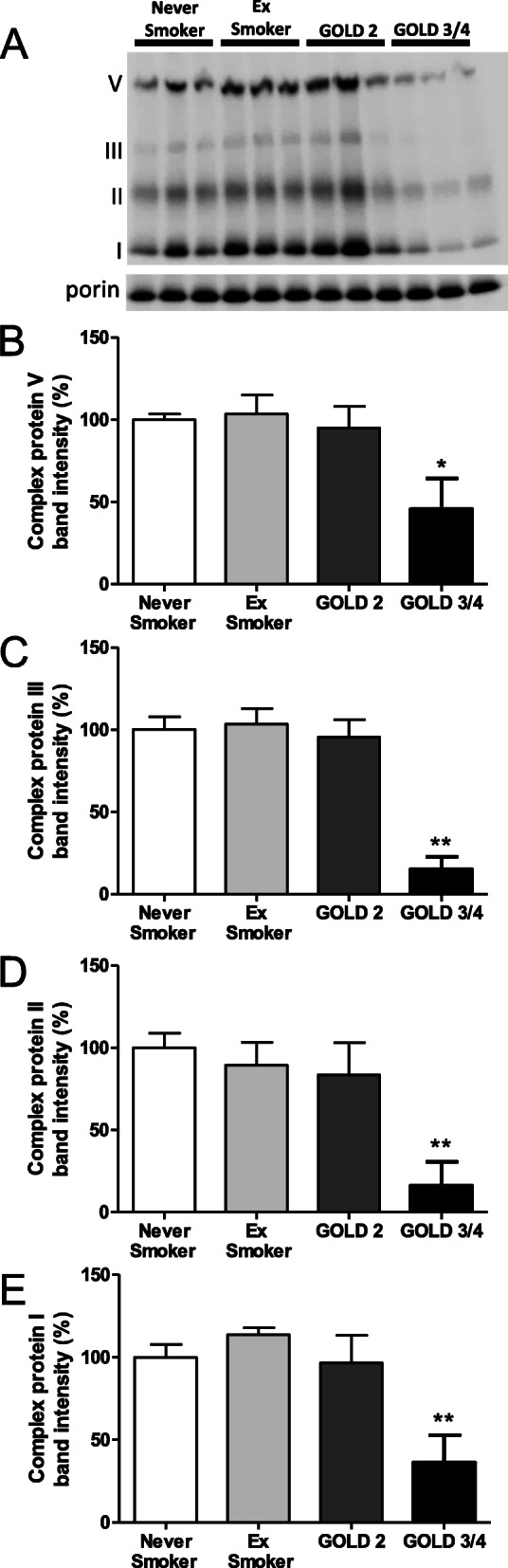
ETC complex protein expression levels in quadriceps muscle. (**a**) Representative Western blot of mitochondrial electron transport chain (ETC) complex proteins from quadriceps muscle of 3 never-smokers, 3 ex-smokers, 3 GOLD Grade 2 COPD patients and 3 GOLD Grade 3/4 COPD patients. (**b**) Levels of ETC complex proteins V, (**c**) levels of ETC complex protein III, (**d**) levels of ETC complex protein II, (**e**) levels of ETC complex protein I; Healthy never-smoker (n = 5), ex-smoker (n = 4), COPD Grade 2 patients (n = 6), COPD Grade 3/4 (n = 6). Data is expressed as mean ± SEM; *P < 0.05 and **P < 0.01, Mann-Whitney test

There was no difference in the level of ETC complex proteins I, II, III and V in the quadriceps of never-smokers, ex-smokers and GOLD Grade 2 COPD subjects (Fig. 6 a-e). However, there was a significant reduction in the level of these ETC complex proteins in GOLD Grade 3/4 COPD subjects (Fig. 6a-e). There was a significant correlation between ΔΨm and the levels of mitochondrial ETC complex proteins I and II in muscle (*r* = 0.70; *p* < 0.01; *r* = 0.56, *p* < 0.05, respectively; Fig. 4c &d).

### Mitochondrial function and ROS levels between bronchial and muscle biopsies

There was no correlation between ΔΨm of mitochondria of bronchial and quadriceps biopsies, but mitochondrial and cellular ROS levels did correlate between bronchial and quadriceps biopsies (Table 2).

**Table 2:** Correlation of mitochondrial function and oxidative stress between bronchial and quadriceps biopsies

	Bronchial vs Quadriceps	
	R	*p* value
**Cellular ROS**	0.463	*p* = 0.045
**Mitochondrial ROS**	0.4868	*p* = 0.0345
**ΔΨm**	−0.175	*p* = 0.4736 (NS)

## Discussion

We have shown the presence of mitochondrial dysfunction in the mitochondrial fraction of bronchial biopsy cells in terms of higher levels of mitochondrial ROS and a reduction in ΔΨm in patients with GOLD Grade 2 COPD compared to healthy never-and ex-smokers. This was associated with a reduction in protein expression of electron transfer chain (ETC) complex proteins I, II and V in the mitochondria of patients with COPD, with a reduction in ETC activity as indicated by the impaired ΔΨm. These changes were associated with an increased oxidative stress that is probably resulting from a reduced antioxidant capacity in the cells contained within the biopsy. The ΔΨm generated by the proton pumps (ETC complex proteins I, III and IV) drives ATP production during oxidative phosphorylation. By contrast, in the patients with COPD, concomitant examination of mitochondrial dysfunction of the quadriceps muscle obtained from the same subjects at GOLD Grade 2, we found no evidence of mitochondrial dysfunction with respect to ΔΨm. Although there was an increase in both cellular and mitochondrial ROS levels, this was not associated with any reduction in the mitochondrial ETC complex proteins, particularly of I and II, which were clearly reduced in the mitochondria of the bronchial biopsies. Of greater interest is the observation that in the quadriceps muscle of patients with more severe COPD at GOLD Grade 3/4, there was a considerable loss of ETC complex I, II, III, and V proteins, without any perturbation of ΔΨm but with excess cellular and mitochondrial ROS, similar to that observed for the quadriceps muscle of COPD patients at GOLD Grade 2.

Although we did not obtain bronchial biopsies from the patients at GOLD Grade 3/4, our data indicate that the mitochondrial dysfunctionality in the lung is quite different from that observed in the locomotor quadriceps muscle. There was a significant correlation between the airway ΔΨm and the physiologic measures of airflow obstruction (FEV1% predicted) and transfer factor to carbon monoxide (TLCO_C)_, a measure of emphysema, and measures of exercise capacity such as the maximal oxygen consumption and the distance walked during the 6-min walk test. This supports the possibility that mitochondrial dysfunction in the airways may be a determinant of exercise capacity, which is also dependent on the degree of the airflow obstruction and lung damage. Changes in mitochondrial content, mitochondrial integrity, mitochondrial fusion/fission processes and mitophagy most likely influence these correlations. By contrast, we found no correlation between ΔΨm and ROS levels in quadriceps muscle with quadriceps muscle strength, FEV_1_, TLCOc, 6-min walk distance and peak VO_2_ consumption, supporting the notion that mitochondrial dysfunction in muscle may not be related to exercise capacity.

Mitochondrial dysfunction in terms of enhanced production of mitochondrial ROS, a reduction in oxidative capacity and an increased autophagy and apoptosis of mitochondria has already been reported in locomotor muscle in patients with COPD [15–18]. Understanding the contribution of physical inactivity, or of the disease process of COPD, could be important in determining the degree of mitochondrial dysfunction particularly in those with advanced COPD. Recent evidence suggests that the low muscle oxidative capacity in COPD cannot be explained by physical inactivity alone and is likely driven by an interaction between epigenetic factors [19] and pathophysiological correlates of COPD [13]. Indeed, in a study of patients with COPD compared to healthy individuals who perform a similar amount of physical activity, there was attenuated skeletal muscle mitochondrial density, reduced mitochondrial respiration and increased oxidative stress in the COPD muscle [20]. Furthermore, the fact that we found mitochondrial abnormalities in the airways would also support the possibility that the widespread mitochondrial abnormalities may be secondary to the COPD. This is supported by a recent study that showed that COPD is accompanied by coordinated patterns of transcription in the quadriceps involving the mitochondria and extracellular matrix, that included genes previously implicated in the process of COPD [21].

We presume that the antioxidant potential of the locomotor striated muscle in COPD is adequate to tackle the excess ROS production. Indeed, the SOD2 levels in muscle biopsies were not affected in the GOLD Grade 2 COPD patient group. However, we did observe a small decrease in SOD2 levels in the GOLD Grade 3/4 COPD patient group, but this did not affect the membrane potential probably due to compensatory effect of other antioxidant defence processes. Overall, our observations indicate that the major site of mitochondrial injury in COPD occurs in the airways. The increased oxidative stress is likely related to exposure to cigarette smoke. However, the number of pack-years was similar between the ex-smoker and COPD groups indicating that the presence of the COPD pathology or severity may not be entirely related to amount of smoking. Another important factor is how the lungs respond to the oxidative stress of cigarette smoke. Genetic factors, epigenetic processes and environmental factors are likely to influence this response, but these have not been assessed in this study. Mitochondrial dysfunction in other organs such as locomotor muscle has been attributed to secondary damage due to circulating agents emanating from the damaged lungs. A reduction in mitochondrial ETC complex protein expression particularly of ETC complex I, the NADH coenzyme Q reductase, which is a proton pump, was seen in bronchial but not in muscle mitochondria at GOLD Grade 2 but observed in muscle mitochondria at GOLD Grade 3/4. However, these changes in ETC complex expression were accompanied by a reduction in ΔΨm in the mitochondria from bronchi but not from the quadriceps muscle. The explanation for this is not provided in this study but could relate to the differences in antioxidant defences available in the quadriceps muscle compared to the bronchial biopsy cells. In addition, the metabolic consequences of loss of ETC complex proteins may also be different in these cell types. This difference may indicate that the airways of patients with COPD show a different mitochondrial defect to the one observed in the skeletal muscle. Skeletal muscle may maintain a high mitochondrial membrane potential, despite a reduction in ETC complex expression, due to an increase in the activity of these complexes. Indeed, increased ETC complex I, III and IV activity accompanied by elevated mitochondrial ROS levels has been reported in the skeletal muscle of patients with moderate and severe COPD [22, 23].

One limitation of this study is the use of bronchial and muscle tissues that contain many different cell types such that the results cannot be related to any specific cell type. These results indicate changes at the organ level, and we can only speculate on the cell type that may be driving these differences. Further research is needed to elucidate how the different cellular components in these tissues are able to contribute to the observed effects.

Isolation of mitochondria from biopsies was performed using the douncer method which potentially can cause mechanical damage to the isolated mitochondria. Disruption of tissue and cellular material might lead to loss or alteration of ETC complex proteins or induce ROS production [24]. However, the advantage of the douncer method is that it is a very quick assay method. Analysis of the isolated mitochondria can thus be done very quickly after taking the biopsy from the patient. In the mitochondrial fractions of both the bronchial and quadriceps biopsies, we did not detect ETC complex protein IV that was part of the antibody cocktail used. Complex protein IV might overlap with complex protein I since they are close in size or are sensitive to the reducing step in our protocol. However, we are able to separate and detect these two proteins in mouse lung samples, so we think this might be related to an antibody detection limitation.

The relatively small number of participants in each group, and the lack of airway mitochondrial data in patients with more severe COPD are also limitations of the study. Despite this, we observed clear-cut differences both at the functional level as well as at the level of ETC protein expression.

## Conclusions

In summary, we show, for the first time to our knowledge, that the oxidative stress-induced mitochondrial changes and dysfunction are different between bronchial and quadriceps muscle tissues from the same patient. Whilst the quadriceps muscle in GOLD stage 2 COPD patients is still able to cope with the elevated oxidative stress, the lungs are severely compromised. Another key finding is that the disease progression is different between bronchial and quadriceps compartments with mitochondrial defects only present in GOLD Stage 3/4 quadriceps muscle biopsies. A major difference between the two tissue types is the variety of different cell types present in these tissues. Future research investigating the mechanisms behind the different cellular contributions within each tissue to the development of the COPD and its disease progression will potentially give crucial insights on COPD pathogenesis and disease progression. In addition, the effects of genetic factors, epigenetic processes and the responses to peripheral factors, including inflammatory and oxidative stress-related markers, are likely to be different between the different tissues or even between the different cellular components constituent of each tissue.

We clearly demonstrate that mitochondrial dysfunction in the lungs, but not in quadriceps muscle, is associated with airflow obstruction and reduced exercise capacity in COPD. The mitochondrial events in the quadriceps muscle may be dissociated from those in the lungs.

## Data Availability

Data sharing is not applicable to this article as no datasets were generated or analysed during the current study.

## Abbreviations

6 MW: Six-minute walk distance
ASM: Airway smooth muscle
ATP: Adenosine triphosphate
BMI: Body mass index
CAT score: COPD assessment test score
COPD: Chronic obstructive pulmonary disease
CPET: Cycle-ergometer cardiopulmonary exercise test
CSE: Cigarette smoke extract
CXCL8: C-X-C motif chemokine ligand 8
DCF: 2′-7′-Dichlorofluorescin diacetate
ΔΨm: Mitochondrial membrane potential
ETC: Electron transport chain
FEV1: Forced expiratory volume in 1 s
FVC: Forced vital capacity
GOLD: Global initiative for chronic obstructive lung disease
IL-1β: Interleukin-1β
IL-6: Interleukin-6
JC-1: 5,5′,6,6′-tetrachloro-1,1′,3,3′-tetraethylbenzimidazolylcarbocyanine iodide
mtROS: Mitochondrial reactive oxygen species
QMVC: Quadriceps maximal voluntary contraction
ROS: Reactive oxygen species
SOD2: Superoxide dismutase 2
SPPB: Short physical performance battery
TLCOc: Single-breath transfer factor of the lung for carbon monoxide
VO_2_: Oxygen consumption
